# Cytokine‐centered strategies to boost cancer immunotherapy

**DOI:** 10.1002/1878-0261.13818

**Published:** 2025-02-07

**Authors:** Sara Manzano, María M. Caffarel

**Affiliations:** ^1^ Biogipuzkoa (previously known as Biodonostia) Health Research Institute San Sebastián‐Donostia Spain; ^2^ IKERBASQUE, Basque Foundation for Science Bilbao Spain

**Keywords:** cytokines, immunotherapy, inflammation, predictive biomarkers

## Abstract

Cytokines have gained attention in oncology in recent years, especially in the context of immunotherapy. Although immune checkpoint inhibitors (ICIs) have revolutionized the treatment of cancer, there are still some challenges to be faced, such as the lack of predictive biomarkers as well as the emergence of resistance and their severe side effects. In this Viewpoint, we discuss the potential of cytokines, the soluble mediators of cancer‐associated inflammation, in immunotherapy. Indeed, both the activation and inhibition of cytokines have been suggested as potential strategies to overcome immunotherapy resistance. In addition, serum levels of certain cytokines can predict response to immunotherapy, and cytokine inhibition could also contribute to prevent side effects induced by ICIs. Thus, although further research is still required, data support that cytokine‐based therapies could be an attractive therapeutic strategy for cancer patients treated with immunotherapy in the near future.

## Graphical abstract

In this Viewpoint article, we discuss the three main cytokine‐centered approaches that may have an impact in the context of cancer immunotherapy: (1) modulation of cytokines to increase the efficacy of immune checkpoint inhibitors (ICIs), (2) cytokine inhibition to decrease immunotherapy‐associated side effects, and (3) potential of cytokines as biomarkers of immunotherapy response.

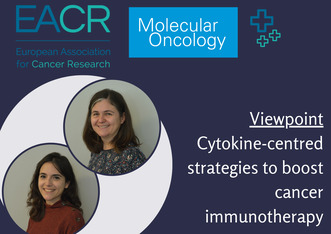

**Abbreviations**
CFS: colony‐stimulating factors
ICI: immune checkpoint inhibitor
IFN: interferon
IL: interleukin
IL6R: interleukin 6 receptor
OSM: oncostatin M
OSMR: OSM receptor
TGF: transforming growth factor
TME: tumor microenvironment
TNF: tumor necrosis factor

## 1 Cancer inflammation and immunotherapies

The important role of inflammation in cancer progression has been known for more than 150 years, but it is still debatable whether it is targetable in the clinic. In the 21st century, two main events have drawn the focus of cancer research to this topic: the inclusion of inflammation as a hallmark of cancer and the change in the paradigm of cancer treatment driven by immunotherapy [1]. Although multiple anticancer immunotherapies are currently being developed, immune checkpoint inhibitors (ICIs), such as antibodies targeting the PD1/PD‐L1 axis, are the most used agents to treat solid cancers. In spite of the revolution driven by ICIs, certain challenges still need to be addressed to expand their benefits [1]: (a) There is a lack of predictive biomarkers, meaning PD‐L1 quantification is clearly insufficient in most solid tumors; (b) the mechanisms driving resistance to ICIs are incompletely understood, and these are clearly influenced by the dynamic interactions of the tumor microenvironment (TME); (c) adverse secondary systemic effects are still evident in patients, highlighting room for improvement in terms of their safety potential.

Although different strategies to target cancer inflammation in the context of immunotherapy have been suggested [1], we consider cytokines to be promising candidate targets to boost the efficacy of immunotherapies in solid tumors. This is mainly due to the role of cytokines controlling the dynamic nature of the TME and their capacity to regulate and recapitulate both the local and systemic effects of cancer‐associated inflammation.

## 2 Cytokines are master regulators of inflammation

**Fig. 1:**
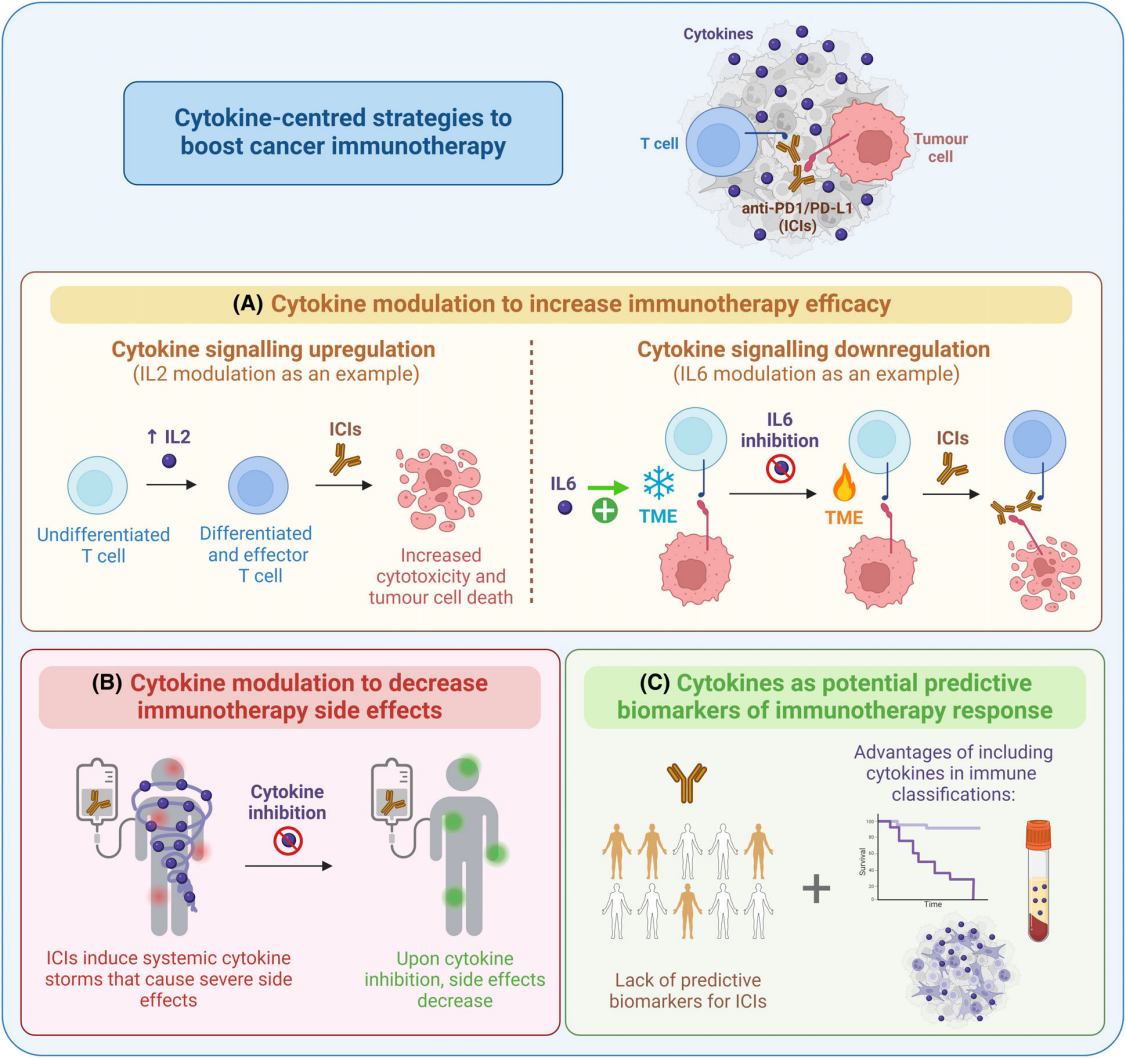
Cytokine‐centered strategies to boost cancer immunotherapy. In the context of immunotherapy, three main cytokine‐centered approaches may have an impact in the clinic. (A) Cytokine modulation has been explored as a therapeutic approach to increase the efficacy of immune checkpoint inhibitors (ICIs). For example, interleukin‐2 (IL2) (*left panel*) promotes the effector capacity of T cells, while interleukin‐6 (IL6) inhibition (*right panel*) decreases immunosuppression in the tumor microenvironment (TME). (B) Immunotherapy induces cytokine storms that threaten the patients' quality of life. Inhibition of cytokines prevents these immune‐related secondary effects. (C) In recent years, different immune‐based classifications are being developed to overcome the lack of predictive biomarkers of immunotherapy and cytokines should be included in these new immune taxonomies of cancer patients. Figure created with https://BioRender.com.

Cytokines are secreted proteins that mediate the communication between immune, stromal and cancer cells in the TME [2]. They are synthesized and recognized by different cell subpopulations, both under physiological and pathological conditions. Cytokines are the main regulators of inflammation, where some cytokines are associated with the promotion of inflammation, while others present anti‐inflammatory properties. There are different families of cytokines: interleukins (ILs), chemokines, adipokines, transforming growth factors (TGFs), tumor necrosis factors (TNFs), colony‐stimulating factors (CSFs), and interferons (IFNs) [2]. The variety of signals or ligands (almost 100 different cytokines in humans), cell types (immune and nonimmune cells), downstream signaling pathways (STATs, NFκB, or MAPKs transcription factors, among others) and negative regulatory mechanisms (modulating signal intensity and duration, such as soluble receptors), together with their capacity to act cooperatively in loops, notably increase the complexity of these cytokine networks [3]. Unraveling this complexity in the context of cancer has been the focus of many laboratories in the world, including ours, in the last years. It is now clear that cytokines are regulated in the TME and in cancer patients' serum, they play a role in all steps of tumor progression, and they can modulate the response to treatment [4, 5, 6, 7, 8]. Recent literature supports that cytokines should be considered in the context of cancer immunotherapy at three levels: as therapeutic targets to (a) decrease resistance to ICIs and (b) reduce ICIs‐related side effects, and (c) as predictive biomarkers of response to immunotherapy (Fig. 1).

## 3 Therapeutic modulation of cytokines

The therapeutic modulation of cytokines has been largely explored, not only in the context of immunotherapy in oncology [1, 2, 9], but mostly for different inflammatory diseases such as psoriatic arthritis, inflammatory bowel disease, or Crohn's disease. Several cytokine inhibitors (antibodies and small molecules) have been clinically evaluated for their ability to inhibit the cytokine storm driving the inflammatory process. Similarly, dysregulation of cytokine loops occurs as an inadvertent effect of cancer immunotherapy and different anticytokine strategies have been explored to block immune‐related adverse events. For example, IL6 receptor (IL6R) inhibition alleviates autoimmunity in patients treated with ICIs [10].

In addition, cytokines can be modulated to potentiate the efficacy of immunotherapies. IL2 is a clear example of how the upregulation of certain cytokines (particularly, antitumor, or immunostimulatory cytokines) can prompt immunotherapy efficacy. It is known that IL2 drives terminal differentiation and promotes effector functions in T cells and, consequently, combination of IL2 with ICIs stimulates effector T cells by two different mechanisms [11].

Inhibition of immunosuppressive and/or tumor‐promoting cytokines is also a very interesting approach to increase immunotherapy efficacy. Our interest in this approach is based on the fact that this strategy may overcome side effects of immunotherapy while also increasing the response to immunotherapy through the same therapeutic combination [10]. This strategy could mostly be applied to IL6 and IL1 cytokine subfamilies [2, 9] where it has been demonstrated that upregulation of these cytokines mediates immunotherapy resistance. Thus, inhibition of certain ILs turns ‘cold tumours’ (that lack effector immune cells and present an immunosuppressive TME) into ‘hot tumours’ (in which infiltrated effector immune cells are in close contact with tumor cells and the antitumor function of immune cells is prompted) [5]. For example, in non‐small cell lung cancer, there are ongoing clinical trials inhibiting IL6, LIF, or IL1β (NCT04691817, NCT05061550, and NCT03631199, respectively) to address immunotherapy resistance.

## 4 Challenges to implement cytokine‐based therapies in the clinic

Nevertheless, some challenges need to be addressed before the full implementation of cytokine‐targeting drugs in oncology and other diseases [1, 2]. One is the wide range of cytokine functions, with the same cytokine often having different effects locally compared to the systemic level. Another difficulty is the cooperation of cytokines through intricate signal networks, their redundancy in functions, and the existence of compensatory mechanisms [3, 12]. Thus, it is important to identify the master cytokine regulators in each cancer context and to design strategies to specifically inhibit them. For example, we discovered that oncostatin M (OSM), a member of IL6 subfamily, drives breast cancer progression by modulation of the TME and the activation of cytokine‐mediated protumour inflammatory networks [13]. Thus, it is essential to study the cytokine networks governing the TME of different cancer types in detail to uncover the master regulators of the tumor‐associated inflammation and to detect therapeutic windows to increase the ratio of response‐to‐toxicity by cytokine‐centered therapies.

## 5 Cytokines as predictive biomarkers for immunotherapy

Finally, cytokines are potential predictive biomarkers for immunotherapy. There is a generalized lack of predictive biomarkers for ICIs, PD‐L1 being the most used in the case of anti‐PD1/PD‐L1 treatments although under some controversies [1]. Recently, immune‐based patient classifications have emerged as potentially useful predictive biomarkers for immunotherapy [14]. As cytokines are the main mediators of inflammation, we consider their integration in these immune classifications as absolutely necessary. Moreover, some cytokines have already been identified as predictive biomarkers of immunotherapy, such as plasma/serum IL6 levels in melanoma, non‐small cell lung cancer, kidney, breast, and bladder cancer patients treated with ICIs [9] or baseline serum IL‐8 levels in pancreatic cancer patients undergoing combined chemotherapy and immunotherapy [15]. Moreover, it is important to highlight that cytokines have the advantage of being circulating biomarkers: minimally invasive, nonexpensive, and perfect candidates to temporarily monitor the dynamic changes occurring in the TME [2, 4, 7].

Nevertheless, some questions remain unanswered. Are changes in cytokine levels in serum an accurate readout of their regulation in the TME? How do current treatments affect the secretion of cytokines? Which is the most clinically relevant method to measure cytokines? Novel multiplex techniques have emerged in recent years, allowing the quantification of multiple cytokines in low sample volumes, with low hands‐on‐time and in low concentration ranges (pg/mL), although they have not been applied yet to clinic workflows. Thus, all these points require further investigation.

## 6 Future directions and conclusions

In conclusion, cytokines are the main mediators of tumor‐associated inflammation and they play an essential role in response to ICIs and as modulators of ICI side effects. Moreover, as cytokines comprise secreted proteins dynamically regulated in the TME, they also have a potential as predictive circulating biomarkers. Although there is still a long way to go before cytokine‐based therapies become a reality for patients with solid tumors, currently available data support their great potential and we envision very active clinical research in these areas in following years.

## Conflict of interest

The authors declare no conflict of interests.
